# Interaction of Pyrrolobenzodiazepine (PBD) Ligands with Parallel Intermolecular G-Quadruplex Complex Using Spectroscopy and ESI-MS

**DOI:** 10.1371/journal.pone.0035920

**Published:** 2012-04-27

**Authors:** Gajjela Raju, Ragampeta Srinivas, Vangala Santhosh Reddy, Mohammed M. Idris, Ahmed Kamal, Narayana Nagesh

**Affiliations:** 1 National Centre for Mass Spectrometry, Indian Institute of Chemical Technology, Hyderabad, India; 2 Division of Organic Chemistry, Indian Institute of Chemical Technology, Hyderabad, India; 3 Centre for Cellular and Molecular Biology, Hyderabad, India; University of Connecticut, United States of America

## Abstract

Studies on ligand interaction with quadruplex DNA, and their role in stabilizing the complex at concentration prevailing under physiological condition, has attained high interest. Electrospray ionization mass spectrometry (ESI-MS) and spectroscopic studies in solution were used to evaluate the interaction of PBD and TMPyP4 ligands, stoichiometry and selectivity to G-quadruplex DNA. Two synthetic ligands from PBD family, namely pyrene-linked pyrrolo[2,1-c][1,4]benzodiazepine hybrid (PBD1), mixed imine-amide pyrrolobenzodiazepine dimer (PBD2) and 5,10,15,20-tetrakis(N-methyl-4-pyridyl)porphyrin (TMPyP4) were studied. G-rich single-stranded oligonucleotide d(5′GGGGTTGGGG3′) designated as d(T_2_G_8_), from the telomeric region of *Tetrahymena Glaucoma*, was considered for the interaction with ligands. ESI-MS and spectroscopic methods *viz*., circular dichroism (CD), UV-Visible, and fluorescence were employed to investigate the G-quadruplex structures formed by d(T_2_G_8_) sequence and its interaction with PBD and TMPyP4 ligands. From ESI-MS spectra, it is evident that the majority of quadruplexes exist as d(T_2_G_8_)_2_ and d(T_2_G_8_)_4_ forms possessing two to ten cations in the centre, thereby stabilizing the complex. CD band of PBD1 and PBD2 showed hypo and hyperchromicity, on interaction with quadruplex DNA, indicating unfolding and stabilization of quadruplex DNA complex, respectively. UV-Visible and fluorescence experiments suggest that PBD1 bind externally where as PBD2 intercalate moderately and bind externally to G-quadruplex DNA. Further, melting experiments using SYBR Green indicate that PBD1 unfolds and PBD2 stabilizes the G-quadruplex complex. ITC experiments using d(T_2_G_8_) quadruplex with PBD ligands reveal that PBD1 and PBD2 prefer external/loop binding and external/intercalative binding to quadruplex DNA, respectively. From experimental results it is clear that the interaction of PBD2 and TMPyP4 impart higher stability to the quadruplex complex.

## Introduction

Ligands containing nitrogen atoms such as azido groups or triazole ring systems and other related functionalities or scaffolds were well known for their therapeutic activity [1], [2]. Number of molecules of this type have been previously reported to interact with G-quadruplex DNA and stabilizes them [3], [4]. Considering the importance of nitrogen containing ligands, couple of PBD based ligands have been selected and examined for their ability to interact and stabilize G-quadruplex DNA. Pyrrolo[2,1-*c*][1,4]benzodiazepine (PBD) ligands belong to the family of naturally occurring antibiotics originated from *Streptomyces* species [5], that exert their cytotoxic activity by binding covalently between C11-position of the PBD and C2-amino group of the guanine residues with in the minor groove of DNA and typical examples of which include DC-81, anthramycin, tomaymycin, sibiromycin and neothramycins [6]. Moreover, PBD ligands bind to DNA sequence selectively and have potential not only as antitumor agents but also as gene regulators and probes of DNA structure [7]. Recently, number of PBD dimers as well as PBD conjugates have been designed and synthesized that exhibit promising anticancer activity with remarkable DNA binding ability [8]–[10]. In addition, PBD dimers which were designed and synthesized by joining two PBD subunits via flexible linkers have been shown to form non distorting interstrand cross-links within the minor groove of DNA and increase in the chain length of the linker in PBD dimers significantly increases the efficiency as anti tumor drugs [11]–[13]. Rettig *et al*., has recently shown by NMR and spectroscopic studies that PBD hybrids bind strongly to the minor groove of the DNA duplex with the formation of a covalent bond between the PBD moiety and an exocyclic guanine amino group 14–16. Various porphyrin-based ligands have been extensively studied for quadruplex interaction and binding, since they can inhibit the activity of telomerase upon binding to human telomeric G-quadruplex DNA [17], [18]. The size of porphyrin ring is similar to that of guanine quartet, hence the stability of G-quadruplex DNA by porphyrin was due to π-π stacking interaction between the porphyrin ring and guanine quartet. Even though it was known that planar porphyrin ligands bind tightly to G-quadruplex structure, there is still some controversy regarding the specific site and molecular nature of their binding [19], [20].

**Figure 1:**
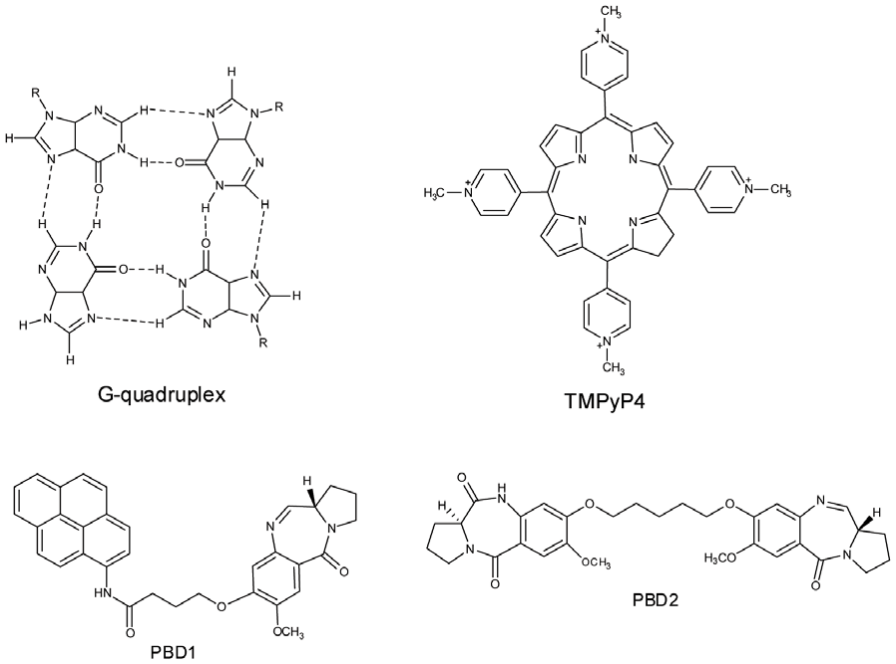
Structure of G-quadruplex, TMPyP4, PBD1 and PBD2.

G-quadruplex (or) G4-DNA structures were composed of stacked tetrads, each having planar association of four guanines arranged in a cyclic manner connected by Hoogsteen hydrogen bonds [21], [22]. Formation of G-quadruplex requires presence of monovalent cations like K^+^, NH_4_^+^ and Na^+^ at the centre [23], [24]. G-rich sequences with the potential to form quadruplex structure were common in genomic DNA, and these have been identified in several biologically important regions, such as the telomeric ends of chromosomes [25], oncogene promoters [26], [27], or immunoglobulin heavy chain switch regions [28]. G-quadruplexes can fold into various conformations where the strands were oriented either in parallel or antiparallel forms and can fold either intra or inter molecularly. Due to the increased interest in quadruplex structures, their interaction with unique ligands which could bind and stabilize them was tested and such ligands could be used as potential anticancer agents, diagnostic tools, and molecular probes. Small organic molecules have been proposed to interact noncovalently with G-quadruplex through tetrad stacking, groove binding, loop binding, and intercalation between the two G-tetrads. Recently, several synthetic molecules were tested for their interaction with quadruplex DNA. Most successful among them were cationic porphyrins [19], [20], acridine derivatives [29], ethidium derivatives [30], anthraquinone derivatives [31], perylene derivatives [32], and telomestatin [33], these have been explored for their ability to bind to quadruplexes and inhibit telomerase activity. Attempts were made from our laboratory in search of suitable ligands which would interact with quadruplex and stabilize [19], [20], [29], [34], [35]. In this context, the present work was designed to study the interaction between d(T_2_G_8_) and PBD ligands as well as TMPyP4. The structure of ligands and G-quadruplex complex were shown in figure 1.

Electrospray ionization mass spectrometry (ESI-MS) has been used as a powerful technique to examine interactions between small ligands and nucleic acids as a result of its low sample consumption, sensitivity and lesser analysis time, making it adaptable to high throughput screening techniques [36]. ESI is a suitable technique which gently lifts the non-covalent complex in the gas phase while keeping the binding interactions intact. Earlier much work has been carried out to study the non-covalent interaction of different ligands with quadruplex DNA [35]–[37]. In the present study, electrospray ionization mass spectrometry and spectroscopic methods *viz.*, UV-Visible, fluorescence, and circular dichroism (CD) have been used to examine the interaction of therapeutically potential ligands like PBD (PBD1 and PBD2) and TMPyP4 with the G-quadruplex formed by d(T_2_G_8_). Throughout the study, interaction with TMPyP4 was considered as a control, as huge data is available on its interaction with quadruplex DNA.

## Materials and Methods

The telomeric region of *Tetrahymena Glaucoma* contains stretches of 5′ TTGGGG 3′ sequences and they were reported to form G-quadruplex complex [23], [38]. Considering this, an oligonucleotide d(5′ GGGGTTGGGG 3′) or d(T_2_G_8_), with a molecular weight of 3180.109, was synthesized using ABI 394 DNA synthesizer and purified by RP-HPLC. 1 mM stock solution of d(T_2_G_8_) was prepared in 100 mM KBPS buffer (30 mM Potassium Phosphate, pH 7.0, with 100 mM KCl, pH 7.0). The oligonucleotide solution was heated to 90°C and slowly cooled to room temperature. DNA samples were dialyzed (1000 molecular weight cutoff membrane) against two changes of buffer (1L, 24 hours each) at 4°C. The DNA solutions were diluted before each experiment to obtain a final quadruplex concentration of 5 µM for CD, UV-Visible, and fluorescence experiments and 10 nM for ESI-MS analysis. For ESI-MS experiments, as ammonium acetate buffer was volatile, analysis of G-quadruplex-ligand complexes were performed in this buffer in order to obtain clean spectra [39] and 10% methanol was used to obtain good spray.

The cationic porphyrin, TMPyP4, was purchased from Frontier Scientific (Logan, Utah, USA) and used without further purification. The stock solution of TMPyP4 was prepared by dissolving weighed amount of ligand in sterile milli Q water. The concentration of TMPyP4 drug was calculated using the molar extinction coefficient €_424_ *= *2.26×10^5^ M*^−^* ^1^Cm*^−^* ^1^. Synthesis of pyrrolobenzodiazepine (PBD) ligands was carried out using the protocol mentioned in the literature [40], [41]. Stock solutions (1 mM) of these ligands were prepared by dissolving weighed amount of each ligand in methanol: water (1∶1).

### Mass spectrometry

Electrospray ionization (ESI) mass spectra were recorded using Exactive Orbitrap mass spectrometer (Thermo Scientific, USA) in negative ion mode. Data was acquired using Xcalibur software (Thermo Scientific). The source conditions maintained were; sheath gas (N_2_) pressure, 35 psi; aux gas pressure, 5 psi; capillary temperature, 120°C; capillary voltage, −50.0 V; tube lens offset voltage, −60 V; skimmer voltage, −40 V; vaporizer temperature, 50°C. Scanning parameters were; higher energy collisional induced dissociation (HCD) gas, off; resolution, enhanced; microscans, 1; lock masses, off, AGC target, balanced and maximum injection time, 200 ms. For ESI experiments, G-quadruplex and ligands concentration was maintained at 10 nM. All the sample solutions were infused into the ESI source at a flow rate of 5 µL/min by using instrument's syringe pump.

### Circular Dichroism spectroscopy

Circular dichroism (CD) experiments were performed using a JASCO 815 CD spectropolarimeter (Jasco, Tokyo, Japan). Quadruplex DNA solution was prepared in 100 mM KBPS buffer (30 mM Potassium Phosphate, pH 7.0, with 100 mM KCl, pH 7.0). G-quadruplex DNA concentration was maintained at 5.0 µM and 1.0 µM, 5.0 µM of each ligand were added. The CD spectra were recorded from 200 to 500 nm in 1 mm path length cuvette. Spectra were averaged over 3 scans, which were recorded at 100 nm/min with a response time of 1 s and a band width of 1 nm.

### Fluorescence spectroscopy

Fluorescence emission spectra were measured at 25°C with Hitachi F4500 spectrofluori- meter (Maryland, USA) using a 1 cm path length quartz cuvette. Quartz cuvettes was thoroughly washed with distilled water and dilute nitric acid (approximately 0.1 N, nitric acid) to minimize nonspecific binding of the ligands to the surface of the cuvette. Throughout the fluorescence experiment, the concentration of ligands were kept constant (5 µM) and titrated with 5 µL of 5 µM G-quadruplex DNA each time. TMPyP4 was excited at 433 nm and emission spectra for each titration were collected from 600 to 800 nm. Both PBD1 and PBD2 ligands were excited at 240 nm, and emission spectra were recorded from 350 nm to 480 nm for PBD1 and 300 nm to 550 nm for PBD2.

### UV-Vis absorption spectroscopy

Absorption spectra were recorded using ABI Lambda Spectrophotometer (Waltham, MA, USA) at 25°C. Experiments were carried out in polystyrene cuvettes to minimize binding of ligands to the surface of the cuvettes. 5 µM of TMPyP4 stock solution was prepared in milliQ water. 5 µM of PBDs solution was prepared in methanol and water (1∶1) and 5 µM of G-quadruplex DNA in 100 mM KBPS buffer. Each ligand (1 mL) was taken in a 1 cm path length cuvette and absorption spectra were recorded after a step wise addition of 5 µL of G-quadruplex DNA ranging from 350 nm to 500 nm. All the solutions used were freshly prepared before starting the experiment. Sample solutions were monitored until equilibrium was reached, as evidenced by constant absorbance readings.

### Quadruplex DNA melting analysis using SYBR Green

To further corroborate the binding and stabilizing ability of the ligands with quadruplex DNA, melting curve analysis of G-quadruplex DNA with and without PBD ligands based on the principle of DNA-DNA relatedness assay using SYBR green (SG) was performed [42], [43]. Quadruplex DNA provides binding grooves to SYBR green molecules. Denaturation of the quadruplex DNA results in the release of SYBR green molecules into the surrounding solvent. 5 µM of quadruplex DNA with and without ligands were mixed with 10 µl of 1× SYBR green and were made to denature at a temperature gradient of 4°C to 94°C with a ramp of 0.2°C per minute in the real time PCR machine (Eppendorf Real Plex, Hamburg, Germany). Melting curve due to the dissociation of quadruplex DNA complex was analyzed and T_m_ was calculated.

### Isothermal titration calorimetry

ITC experiments were performed using Microcal VP-ITC (Northampton, MA, USA). All the ITC experiments were done by filling the ITC cell with approximately 1.5 mL of d(T_2_G_8_) G-quadruplex DNA solution and its concentration was maintained at 20 µM and ligand concentration in the titration syringe was maintained at 5 µM. Each time 60 injections (5 µL) of PBD were added to ITC cell. Ligand addition to G-quadruplex DNA was made for every 180 seconds. Throughout the ITC experiment, the final dialysate from the appropriate oligonucleotide was used in order to maintain heat of ligand dilution constant. Each ITC experiment was performed in triplicate to avoid errors generated while performing the experiment. The integrated heat/injection data obtained in each ITC titration were fitted with algorithm developed for Mathamatica 5.0 software. In the present study, though the stoichiometry was 1∶1, the data were fitted within the experimental error using two-sites binding model to understand ligand binding affinity towards both the binding sites on quadruplex DNA. As reported earlier [44] equilibrium of macromolecules, like quadruplex DNA with multiple ligand binding sites can be illustrated by two different association constants. Thermodynamic parameters like, ΔG1, ΔG2, ΔH1, ΔH2, −TΔS1, −TΔS2, K1 and K2 were extracted directly from the fits.

## Results and Discussion

### Mass spectrometry

**Figure 2:**
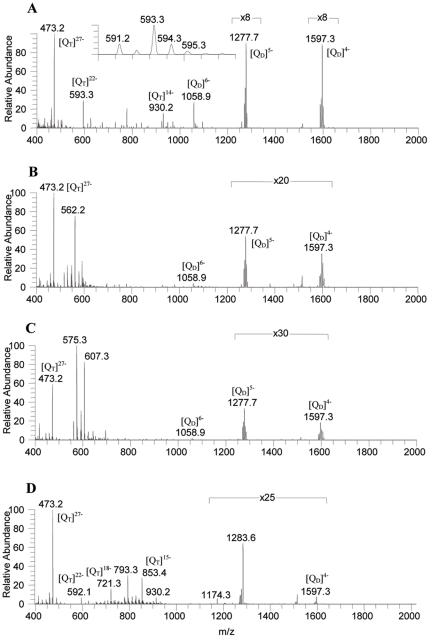
ESI-MS spectra of A d(T_2_G_8_) G-quadruplex, B G-quadruplex with PBD1, C G- quadruplex with PBD2, and D G-quadruplex with TMPyP4 drug.

Electrospray ionization (ESI) is a soft ionization technique which allows detecting intact non-covalent complexes at very low concentration [36]. It is essential to study G-quadruplex-ligand interaction at low concentrations (close to *in vivo* condition), stoichiometry, affinity of each ligand towards quadruplex, and number of cations in each complex. To investigate the interaction of PBDs and TMPyP4 with G-quadruplex DNA, ESI mass spectra of each ligand with G-quadruplex were recorded and the details of mass spectral studies were given in table 1. The ESI-MS spectrum of G-rich single strand d(5′GGGGTTGGGG 3′) or d(T_2_G_8_) in 50 mM ammonium acetate solution shows deprotonated G-quadruplex ions at *m/z* 473 (base peak), 593, 930, 1060, 1278 and *m/z* 1597 with charge states of −27, −22, −14, −6, −5, and −4, respectively (Figure 2a). The ions at *m/z* 473, 593, and 930 correspond to the four-stranded structure d(T_2_G_8_)_4_ with presumably two, nine, and eight potassium cations, respectively ([Q_T_+2K−H]^27−^, [Q_T_+9K−H]^22−^, and [Q_T_+8K−H]^14−^). We speculate that eight potassium cations stabilize eight G-quartets with one more potassium ion placed in the central region of quadruplex DNA, associate with a quartet formed by thymidines. It was shown that like guanines, thymidines also form clusters stabilized by central cations [45]. Formation of *m/z* 1060, 1278 and 1597 ions can be attributed to the two hairpin structures formed by d(T_2_G_8_)_2_ with specifically one and two ammonium adducts, at charge states of −6, −5 and −4, respectively ([Q_D_+NH_4_−3H]^6−^, [Q_D_+2NH_4_−H]^5−^ and [Q_D_+2NH_4_−2H]^4−^). Q_D_ and Q_T_ corresponds to quadruplex in the dimeric (two hairpin structures) and tetrameric forms respectively. Since cations were known to sit between two stacks or in the centre of quadruplex [46], complex formed by d(T_2_G_8_)_2_ and d(T_2_G_8_)_4_ was stabilized by ammonium and potassium cations, respectively, indicate that these ions were placed in the centre of the quadruplex. The ammonium or potassium ions are expected to be readily eliminated if they are non-specifically attached to the DNA phosphate groups.

**Table 1:** ESI-MS spectra of PBDs (PBD1 and PBD2) and TMPyP4 with G-quadruplex DNA with *m/z* (Relative abundances) and stoichiometry.

G-quadruplex	Ligand	*m/z* (Relative abundance)	Charge of the complex	Quadruplex∶Ligand
**d(T_2_G_8_)**	No ligand	473 (100)	[Q_T_+2K−H]^27−^	1∶0
		593 (30)	[Q_T_+9K−H]^22−^	1∶0
		930 (15)	[Q_T_+8K−H]^14−^	1∶0
		1060 (27)	[Q_D_+NH_4_−3H]^6−^	1∶0
		1278 (12)	[Q_D_+2NH_4_−H]^5−^	1∶0
		1597 (11)	[Q_D_+2NH_4_−2H]^4−^	1∶0
**d(T_2_G_8_)**	PBD1	473 (100)	[Q_T_+2K−H]^27−^	1∶0
		516 (15)	[Q_T_+PBD1+5K−H]^26−^	1∶1
		530 (23)	[Q_T_+PBD1+K−H]^25−^	1∶1
		545 (23)	[Q_T_+10K−H]^24−^	1∶0
		562 (75)	[Q_T_+PBD1+7K−H]^24−^	1∶1
		578 (15)	[Q_T_+PBD1+2K−H]^23−^	1∶1
		592 (28)	[Q_T_+8K+NH_4_−H]^22−^	1∶0
		608 (3)	[Q_T_+PBD1+4K−H]^22−^	1∶1
		622 (3)	[Q_T_+9K+NH_4_−H]^21−^	1∶0
		638 (3)	[Q_T_+PBD1+4K−H]^21−^	1∶1
		652 (2)	[Q_T_+9K−H]^20−^	1∶0
		1060 (4)	[Q_D_+NH_4_−3H]^6−^	1∶0
		1277 (2.6)	[Q_D_+2NH_4_−2H]^5−^	1∶0
		1597 (2)	[Q_D_+2NH_4_−2H]^4−^	1∶0
**d(T_2_G_8_)**	PBD2	473 (60)	[Q_T_+2K−H]^27−^	1∶0
		561 (17)	[Q_T_+PBD2+5K−H]^24−^	1∶1
		575 (100)	[Q_T_+PBD2+NH_4_−2H]^23−^	1∶1
		593 (30)	[Q_T_+9K−H]^22−^	1∶0
		607 (82)	[Q_T_+PBD2+2K−H]^22−^	1∶1
		653 (4)	[Q_T_+9K−H]^20−^	1∶0
		667 (4)	[Q_T_+PBD2+2K−H]^20−^	1∶1
		1060 (3)	[Q_D_+NH_4_−4H]^6−^	1∶0
		1278 (1.0)	[Q_D_+2NH_4_−H]^5−^	1∶0
		1597 (0.5)	[Q_D_+2NH_4_−2H]^4−^	1∶0
**d(T_2_G_8_)**	TMPyP4	473 (100)	[Q_T_+2K−H]^27−^	1∶0
		592 (5)	[Q_T_+8K+NH_4_−H]^22−^	1∶0
		721 (15)	[Q_T_+7K−H]^18−^	1∶0
		793 (30)	[Q_T_+TMPyP_4_+6K−2H]^18−^	1∶1
		853 (27)	[Q_T_+2K+NH_4_−H]^15−^	1∶0
		930 (7)	[Q_T_+8K−H]^14−^	1∶0
		1174 (0.3)	[Q_T_+TMPyP_4_+NH_4_−H]^12−^	1∶1
		1284 (2.6)	[Q_T_+TMPyP_4_+3NH_4_−H]^11−^	1∶1
		1597 (0.4)	[Q_D_+2NH_4_−2H]^4−^	1∶0

The ESI-MS spectrum of G-quadruplex with PBD1 was shown in figure 2b. The spectrum exhibits the ions at *m/z* 473, 516, 530, 545, 562, 578, 592, 608, 622, 638, 652, 1060, 1277, and 1597. It has been observed that the intensities of lower charged species at *m/z* 1060 ([Q_D_+NH_4_−3H]^6−^), 1277 ([Q_D_+2NH_4_−2H]^5−^) and *m/z* 1597 ([Q_D_+2NH_4_−2H]^4−^) decreased after the addition of PBD1. The peaks at *m/z* 473, 545, 592, 622, and 652 can be attributed to ([Q_T_+2K−H]^27−^), ([Q_T_+10K−H]^24−^), ([Q_T_+8K+NH_4_−H]^22−^), ([Q_T_+9K+NH_4_−2H]^21−^) and ([Q_T_+9K−H]^20−^), respectively. Whereas, the peaks at *m/z* 516, 530, 562, 578, 608, and 638 correspond to ([Q_T_+PBD1+5K−H]^26−^), ([Q_T_+PBD1+K−H]^25−^), ([Q_T_+PBD1+7K−H]^24−^), ([Q_T_+PBD1+2K−H]^23−^), ([Q_T_+PBD1+4K−H]^22−^) and ([Q_T_+PBD1+4K−H]^21−^), respectively formed with the interaction of PBD1. Figure 2c shows the ESI-MS spectrum of G-quadruplex with PBD2. The spectrum exhibits the peaks at *m/z* 473, 561, 575, 593, 607, 653, 667, 1060, 1278, and 1597. It has been observed that after the addition of PBD2, the intensities of *m/z* 1060 ([Q_D_+NH_4_−4H]^6−^), 1277 ([Q_D_+2NH_4_−2H]^5−^) and 1597 ([Q_D_+2NH_4_−2H]^4−^) decreased. The ions at *m/z* 473, 593 and 653 can be attributed to ([Q_T_+2K−H]^27−^), ([Q_T_+9K−H]^22−^) and ([Q_T_+9K−H]^20−^), respectively. The formation of ions at *m/z* 561, 575, 607 and 667 corresponding to ([Q_T_+PBD2+5K−H]^24−^), ([Q_T_+PBD2+NH_4_−3H]^23−^), ([Q_T_+PBD2+2K−H]^22−^) and ([Q_T_+PBD2+2K−H]^20−^), respectively indicate that the interaction of PBD2 with the G-quadruplex DNA. Figure 2d shows the ESI-MS spectrum of the G-quadruplex with TMPyP4. The spectrum shows the ions at *m/z* 473, 592, 721, 793, 853, 930, 1174, 1284, and 1597. The ions at *m/z* 721, 793, 853, 930, 1174, and 1284 can be attributed to ([Q_T_+7K−H]^18−^), ([Q_T_+TMPyP_4_+6K−2H]^18−^), ([Q_T_+2K+NH_4_−H]^15−^), ([Q_T_+8K−H]^14−^), ([Q_T_+TMPyP_4_+NH_4_−H]^12−^) and ([Q_T_+TMPyP_4_+3NH_4_−H]^11−^), respectively. Occurrence of peaks with *m/z* 793, 1174, and 1284 ions clearly indicate an interaction between TMPyP4 and G-quadruplex DNA. From the present study, it was understood that when TMPyP4 was added to G-quadruplex DNA in 1∶1 molar ratio (10 nM), the relative abundance of peaks, corresponding to the G-quadruplex DNA decreased significantly. This indicates an effective interaction of TMPyP4 and stabilization of d(T_2_G_8_) G-quadruplex. Probably due to lower ammonium ion concentration and formation of secondary structures, the peaks in the lower m/z region (m/z 400–1000) were sharp and intense with low abundance cationic adducts around them. In order to view these peaks clearly with their cationic adducts, the lower m/z region was expanded and shown in (Figure S1, Figure S2, Figure S3, Figure S4).

A parameter, IRa [47], that denotes the relative binding affinity of PBD ligands (PBD1 and PBD2) and TMPyP4 to the d(T_2_G_8_) G-quadruplex DNA, was calculated by using the following equation.
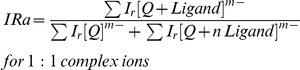



Where ∑ I_r_[Q]^m−^ and ∑ I_r_[Q+Ligand]^m−^ were the total intensities of G-quadruplex and 1∶1 quadruplex complex: ligand, respectively. Table 2 shows the relative binding affinity (IRa) of the ligands with the G-quadruplex. These ligands have different binding affinities for 1∶1 complexes. The IRa values show that the binding affinity of PBD1 was comparatively less than PBD2. This indicates that the interaction of PBD2 with the G-quadruplex DNA is more as compared to the interaction of PBD1 with the G-quadruplex. Intercalation between quadruplex and ligand depends on certain factors like, planarity and orientation of the interacting ligand. PBDs were previously reported to possess planar structure and intercalate to DNA [12], [48]. The relative binding affinity values for PBD1 and PBD2 were found to be 0.45 and 0.67 respectively. This indicates that the binding affinity of PBD ligands was in the order, PBD2>PBD1. TMPyP4 ligand was reported to bind to the G-quadruplex DNA through intercalation, because of its planarity [49].

**Table 2:** IRa values of ligands (PBD1, PBD2, and TMPyP4) with the G-quadruplex DNA.

Ratio of Quadruplex∶Ligand	IRa (Relative binding affinity)
**Q:PBD1 (1∶1)**	0.45
**Q:PBD2 (1∶1)**	0.67
**Q:TMPyP4 (1∶1)**	0.18

### CD spectroscopy

**Figure 3:**
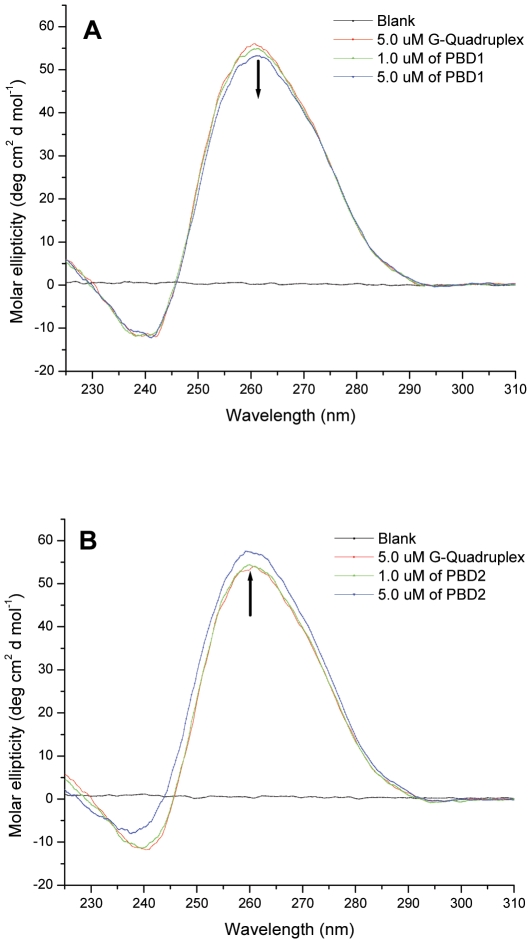
CD spectra of A PBD1on interaction with G-quadruplex and B PBD2 interaction with G-quadruplex in 100 mM KBPS buffer (pH 7.0).

Circular dichroism has been used to understand the changes in the conformation of G-quadruplex on interaction with ligand. Parallel quadruplexes display a positive CD peak around 265 nm and a negative peak at 240 nm, while antiparallel ones exhibit a positive peak at around 295 nm, and a negative peak around 260 nm [50]. CD spectrum of 5.0 µM G-quadruplex with d(T_2_G_8_) showed a prominent positive peak at around 265 nm and a negative peak at 240 nm, indicating the presence of parallel inter-molecular quadruplex conformation in 100 mM KBPS buffer. Figure 3a demonstrates the effect of PBD1 on interaction with d(T_2_G_8_) quadruplex complex. On addition of 1.0 µM and 5.0 µM of PBD1 to G-quadruplex DNA, the peak at 265 nm exhibits hypochromicity and very low negative cotton effect. Hypochromicity of CD band was low and no shift of soret band was observed. The hypochromicity of CD band can be attributed to the partial unfolding of G-quadruplex DNA [51]. Figure 3b shows the CD spectrum of G-quadruplex with PBD2. On addition of 1.0 µM and 5.0 µM of PBD2 to G-quadruplex DNA, hyperchromicity and lesser positive cotton effect was observed. This indicates that PBD2 interacts with the parallel inter-molecular G-quadruplex DNA and stabilizes the complex [52].

The CD spectrum of G-quadruplex DNA with 1.0 µM and 5.0 µM of TMPyP4 exhibits a more hypochromicity, induced CD (ICD) at 463 nm and negative cotton effect probably due to higher intercalation and end loop binding of TMPyP4 to G-quadruplex DNA (spectrum is not shown). Among the porphyrins, TMPyP4 was known to have better interaction with G-quadruplex DNA [19], [20]. From circular dichroism experiments, it was evident that the interaction of the PBD ligands with G-quadruplex DNA was in the order; PBD2>PBD1.

### UV-Visible spectroscopy

**Figure 4:**
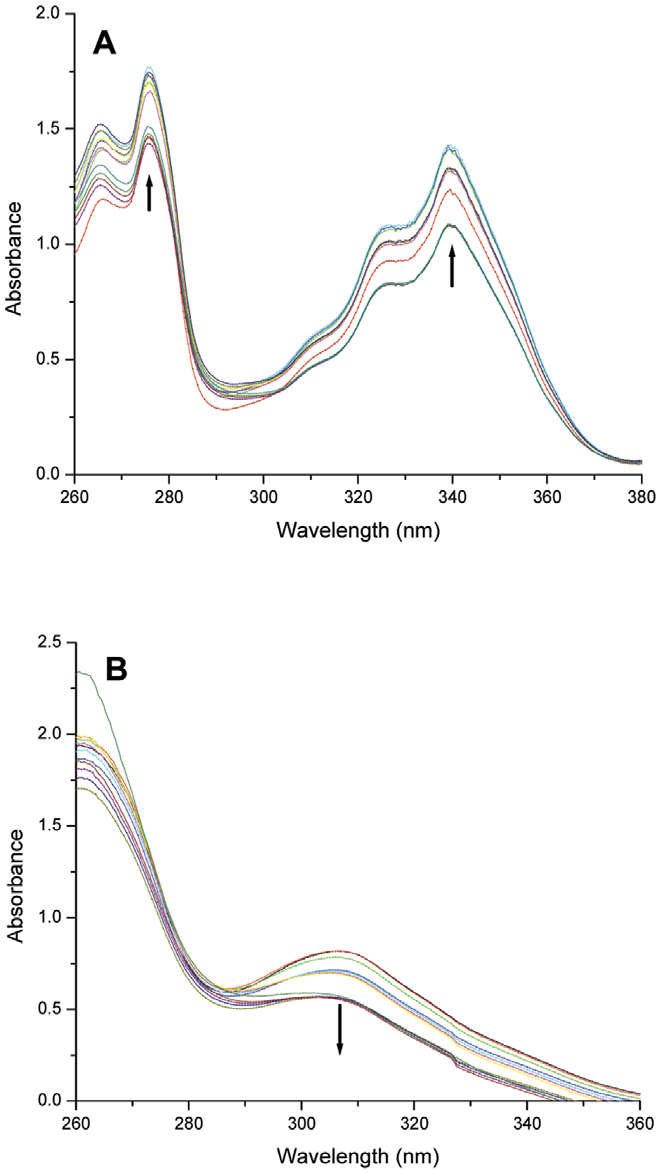
UV-Visible spectra of A G-quadruplex (5 µM) on interaction with PBD1 ligand and B G-quadruplex (5 µM) on interaction with PBD2 ligand.

UV-Visible spectroscopy is an excellent technique to comprehend DNA-ligand binding. To gain insight into the interaction between ligands and the G-quadruplex, the UV-Visible spectra of PBD ligands (PBD1 and PBD2) and TMPyP4 were recorded in the absence and presence of G-quadruplex DNA. The titration was continued until the wavelength and intensity of the absorption band of ligand did not change any more upon three successive additions of G-quadruplex. UV-Vis spectrum of PBD1 was remarkably different, consisting of two absorption peaks at 276 and 339 nm (Figure 4a). The soret band exhibited less hyperchromicity and does not show any shift either from 276 or from 339 nm. No shift of the soret UV-Visible peak and lesser hyperchromicity indicate that interaction of PBD1 with quadruplex DNA takes place through external binding. The isobestic point was observed at around 280 nm. The isobestic point was not a sharp, tight single point. The slight deviation of isobestic point from a single point indicates that the interaction of PBD1 with G-quadruplex DNA occurs through multiple steps.

The UV-Visible spectrum of PBD2 exhibits the soret band at 306 nm (Figure 4b). The soret band shows hypochromicity and does not show any shift. Presence of less intense hypochromic band indicates a mild intercalation of PBD2 with the G-quadruplex DNA. The isobestic point was observed at around 276 nm and the deviation of isobestic point from a single point shows that the interaction of PBD2 with the G-quadruplex DNA takes place through multiple steps. On interaction of d(T_2_G_8_) quadruplex complex to TMPyP4, the porphyrin soret band has shown bathochromic shift of 10 nm and higher percentage of hypochromicity compared to PBD2. This indicates that TMPyP4 has a higher level of interaction (mostly by intercalation) with quadruplex DNA. Higher level of TMPyP4 interaction with quadruplex DNA may be due to planar structure of TMPyP4 molecule compared to PBD2. The UV-Visible data obtained on ligand-quadruplex DNA interaction was shown in table 3. Percentage of hypochromicity for each ligand was calculated by following the procedure described by Keating and Szalai [53]. From the results obtained from the spectroscopic and mass experiments, we speculate that PBD1 bind externally and PBD2 exhibit both modes of interaction (external binding/intercalation) with quadruplex DNA. TMPyP4 interacts more efficiently to the G-quadruplex DNA than PBD ligands.

**Table 3:** UV-Visible spectral titration results for binding of PBD1, PBD2, and TMPyP4 to d(T_2_G_8_) G-quadruplex.

G-quadruplex	Ligand	Soret band shift (nm)	Isobestic point (nm)	% hypochromicity	% hyperchromicity
**d(T_2_G_8_)**	PBD1	No shift	280	-	16.2
**d(T_2_G_8_)**	PBD2	No shift	276	29.5	-
**d(T_2_G_8_)**	TMPyP4	424–434	435	38.2	-

### Fluorescence spectroscopy

**Figure 5:**
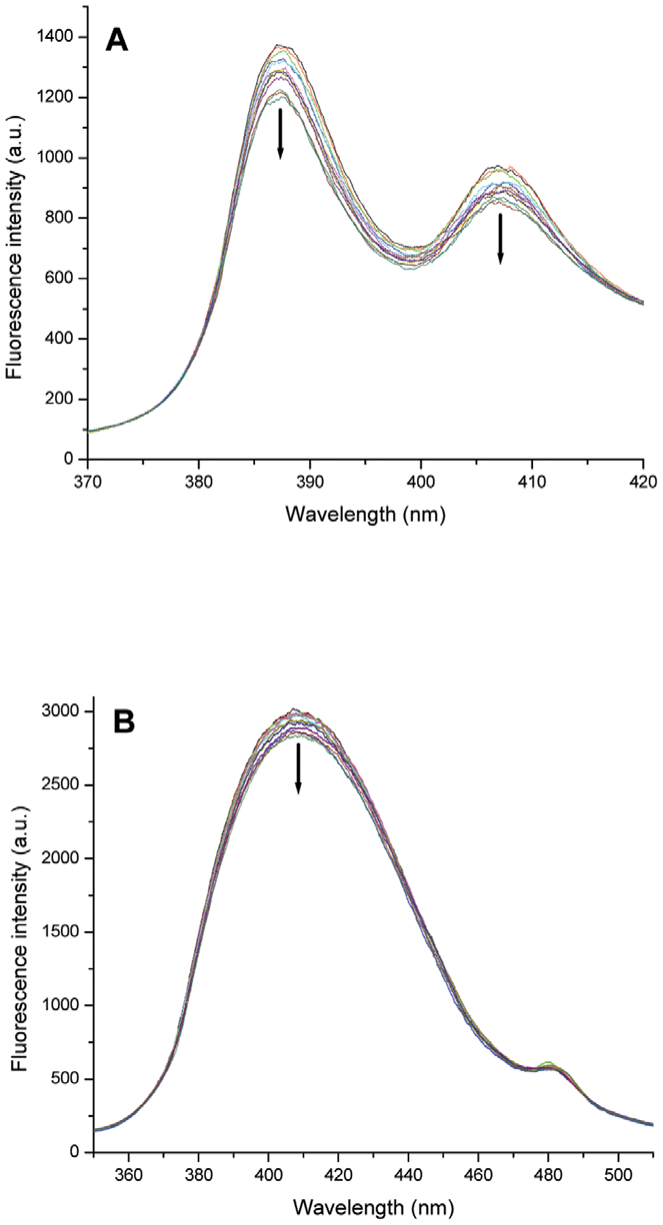
Fluorescence emission spectra of A G-quadruplex on interaction with PBD1 ligand and B G-quadruplex on interaction with PBD2 ligand.

In order to understand the mode of ligand interaction with G-quadruplex DNA fluorescence emission spectra for PBD ligands (PBD1 and PBD2) and TMPyP4 were recorded in the absence and presence of different amounts of G-quadruplex DNA. Figure 5a shows the representative fluorescence spectrum of PBD1 titrated with different amounts of G-quadruplex DNA. Fluorescence emission spectrum with PBD1 shows two peaks corresponding to 387 and 407 nm, which gradually decrease on addition of G-quadruplex. The fluorescence quenching observed for two peaks at 387 nm and 407 nm were found to be 13.13% and 11.03%, respectively. The emission spectrum of PBD2 shows two emission peaks at 410 nm and 482 nm. The fluorescence emission peak at 410 nm slightly decreased on addition of G-quadruplex DNA and its quenching was found to be 4.9%. From figure 5a and 5b it was evident that the fluorescence quantum yield of PBD2 was much higher than PBD1. This may be due to π-π electronic transitions occurring between the ligand and the guanine bases when PBD2 intercalates between two quartets. We speculate that on addition of G-quadruplex DNA, fluorescence quenching of PBD1 and PBD2 occurs due to two reasons, firstly, the PBDs prefers to bind externally to the G-quadruplex and expose to the solvent environment, resulting in the quenching of fluorescence. Secondly, binding of PBD ligands takes place externally due to the smaller size of the quadruplex complex formed by d(T_2_G_8_). Quenching of PBD1 (13.13% and 11.03% for peaks at 387 nm and 407 nm, respectively) was more compared to PBD2 (4.9% for 410 nm peak) on addition of G-quadruplex DNA, indicating the preferential binding of PBD1 to quadruplex DNA, externally. From figure 5b, it was evident that PBD2 fluorescence quantum yield was high and quenching was low. This may be due to moderate intercalation of PBD2 with quadruplex DNA. This is in agreement with UV-Visible experimental results.

The fluorescence emission spectrum of TMPyP4 shows a peak at 780 nm. On addition of G-quadruplex DNA, the fluorescence intensity of TMPyP4 was enhanced to 18.8%. From this, it was evident that fluorescence enhancement of TMPyP4 may be attributed to intercalative and end loop binding [54]. On intercalation, TMPyP4 was sandwiched between two quartets, which shield the ligand from surrounding solvent molecules, resulting in the fluorescence intensity enhancement. From the experimental results, we speculate that PBD1 binds externally and PBD2 moderately intercalates to d(T_2_G_8_) quadruplex DNA. The experimental details obtained from fluorescence titrations were given in table 4. Studies on the interaction of PBD ligands with quadruplex DNA structures formed by various promoter regions of oncogenes are in progress.

**Table 4:** Fluorescence emission titration results for binding of PBD1, PBD2, and TMPyP4 to d(T_2_G_8_) G-quadruplex.

G-quadruplex	Ligand	Soret band (nm)	% of fluorescence quenching	% of fluorescence enhancement
**d(T_2_G_8_)**	PBD1	387	13.13	-
**d(T_2_G_8_)**	PBD1	407	11.03	
**d(T_2_G_8_)**	PBD2	410	4.9	-
**d(T_2_G_8_)**	TMPyP4	780	-	18.79

### Quadruplex DNA melting analysis using SYBR Green

**Figure 6:**
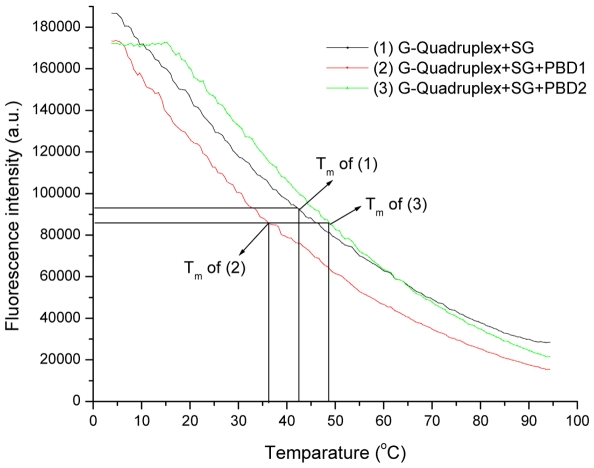
Melting of (1) G-quadruplex using SYBR Green alone, (2) with PBD1, and (3) with PBD2.

From earlier studies, it was known that dsDNA specific dyes like SYBR Green I (SG) exhibits fluorescence via surface or groove binding, when the ratio of base pair to SG molecule complexes exceeds 0.15 [55]. In the present study, stabilization and binding affinity of PBD ligands (PBD1 and PBD2) to G-quadruplex DNA was studied by exploiting the specificity and higher fluorescence of SG on binding to G-quadruplex DNA. On increase of temperature, quadruplex DNA melts to single stranded form releasing the SG molecules into the surrounding environment. This will decrease SG fluorescence. Figure 6 demonstrates the melting profiles of quadruplex DNA alone, quadruplex with PBD1 and PBD2 with SG which were marked 1, 2, and 3 respectively. From the melting profile, it was evident that T_m_ of quadruplex alone, with PBD1 and PBD2 was 43°C, 36°C and 49°C respectively. Lowering of T_m_ after the addition of PBD1 indicates unfolding of quadruplex DNA to single stranded form. About 6°C increase of T_m_ with PBD2 confirms stabilization of G-quadruplex DNA. The results obtained from DNA melting experiments using SG were in agreement with the spectroscopic and ESI-MS studies. The present study was aimed to understand the affinity and the interaction of ligands from a PBD family with quadruplex DNA, a non-canonical DNA structure, presumably involved in transcription regulation; indicate that PBD2 and similar structured ligands might function as an efficient anti-cancer agents.

### Isothermal titration calorimetry

**Figure 7:**
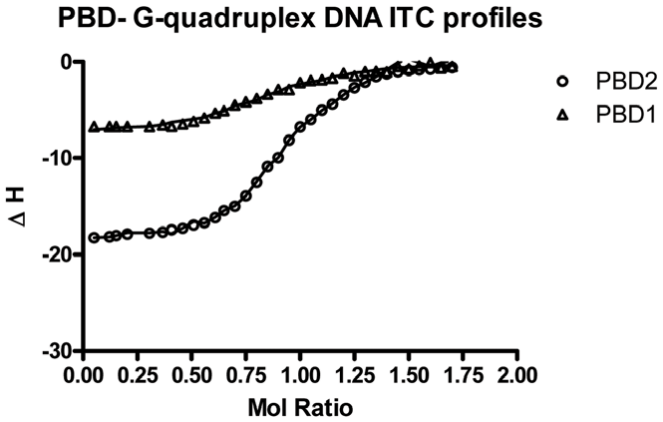
ITC binding isotherms for titration of PBD ligands (PBD1 and PBD2) with G- quadruplex DNA in 100 mM KBPES (pH 7.0) buffer.

Isothermal calorimetry was used as a sensitive and direct micro calorimetric technique with precession for determining the binding affinity, stoichiometry and thermodynamic parameters [56]. Interaction between d(T_2_G_8_) G-quadruplex DNA and PBD ligands (PBD1 and PBD2) was analyzed at 25°C using 100 mM KBPS (pH 7.0) buffer was documented in figure 7 and the results obtained were shown in table 5. From the results, it was clear that PBD2 interaction with quadruplex occurs in two ways. One is a most favorable interaction process (mode-1), which takes place in combination of contribution from enthalpy (−2 to −4 kcal/mol) and significant favorable entropy (−3.0 to −6.0 kcal/mol). The second process (mode-2), which was relatively unfavorable process, proceeds with more significant enthalpy (−5.0 to −7.0 kcal/mol) contri- bution and a smaller entropy range (−1 to −3 kcal/mol). Analyzing the ITC results, the energy profiles show that interaction of PBD1 takes place with one binding mode closer to higher affinity binding (mode-1) of PBD2, considering the relative entropy and enthalpy contribution to the overall binding free energy change.

**Table 5:** Thermodynamic parameters for the interaction of PBD ligands (PBD1 and PBD2) with G-quadruplex determined by ITC at 25°C and pH 7.0.

ITC Derived Thermodynamic Parameters	PBD1-Quadruplex DNA	PBD2-Quadruplex DNA
**K1×10^−8^**	0.0597 (±0.01)	0.0875 (±0.03)
**ΔG1 (kcal/mol)**	−8.73 (±0.02)	−6.35 (±0.01)
**ΔH1 (kcal/mol)**	−2.57(±0.03)	−3.46 (±0.02)
**−TΔS1 (kcal/mol)**	−6.09 (±0.01)	−3.47 (±0.01)
**K2×10^−6^**	0.222(±0.15)	0.483 (±0.11)
**ΔG2 (kcal/mol)**	−4.65 (±0.01)	−3.23 (±0.03)
**ΔH2 (kcal/mol)**	−6.54 (±0.01)	−5.86 (±0.01)
**−TΔS2 (kcal/mol)**	−2.87 (±0.02)	−1.82 (±0.01)

Parameters mentioned above are for a two-sites binding model. The uncertainties mentioned for the two fitting parameters, Ki and ΔHi, were determined from Monte Carlo analysis.

From the ITC results, the swift and higher binding process, which is more entropy driven resembles the binding of ligands to the exterior of DNA. It may be the binding of PBD ligands to the exterior or the two terminal end loops (end staking) of the quadruplex DNA. It may be due to binding of non planar (like PBD1) ligands externally, which cannot interact with quadruplex DNA by staking between the two orderly placed tetrads. The second process is enthalpy driven as the binding of a planar molecule between the two orderly placed tetrads occurs with a typical exothermic enthalpy change due to increased π-π staking interaction between the interacting ligand and the DNA bases [57]–[61]. We speculate that the second process which is significantly enthalpy driven is a more likely an “intercalation process”.

### Conclusions

In this study, electrospray ionization mass spectrometry (ESI-MS), microcalorimetry and spectroscopy have been used to evaluate the stoichiometry, stability, ligand-DNA interaction, and selectivity of PBDs (PBD1 and PBD2) and TMPyP4 to quadruplex DNA. ESI-MS, ITC, UV-Visible and fluorescence experiments indicate that PBD1 exhibits external/end-loop binding whereas PBD2 moderately intercalates/bind externally to quadruplex DNA. CD experimental data demonstrate the existence of parallel inter-molecular G-quadruplex in solution. CD and melting studies with SYBR green reveals that PBD1 unfolds and PBD2 stabilizes the quadruplex DNA.

## Supporting Information

Figure S1 **Expanded ESI-MS spectrum (from m/z 400–1000) of d(T_2_G_8_) G-quadruplex.**
(TIF)

Figure S2 **Expanded ESI-MS spectrum (from m/z 400–1000) of d(T_2_G_8_) G-quadruplex with PBD1. The interaction peaks were marked with “•”.**
(TIF)

Figure S3 **Expanded ESI-MS spectrum (from m/z 400–1000) of d(T_2_G_8_) G-quadruplex with PBD2. The interaction peaks were marked with “♦”.**
(TIF)

Figure S4 **Expanded ESI-MS spectrum (from m/z 400–1000) of d(T_2_G_8_) G-quadruplex with TMPyP4. The interaction peaks were marked with “▪”.**
(TIF)
