# Correction: Interaction of Pyrrolobenzodiazepine (PBD) Ligands with Parallel Intermolecular G-Quadruplex Complex Using Spectroscopy and ESI-MS

**DOI:** 10.1371/annotation/d51e5efe-c697-4400-bac2-67027fbb88b5

**Published:** 2012-06-07

**Authors:** Gajjela Raju, Ragampeta Srinivas, Vangala Santhosh Reddy, Mohammed M. Idris, Ahmed Kamal, Narayana Nagesh

There is missing information in all of the affiliations. They should read:

1. National Centre for Mass Spectrometry, Council of Scientific and Industrial Research (CSIR)-Indian Institute of Chemical Technology, Hyderabad 500007, India.

2. Council of Scientific and Industrial Research (CSIR)-Indian Institute of Chemical Technology, Hyderabad 500007, India.

3. Council of Scientific and Industrial Research (CSIR)-Centre for Cellular and Molecular Biology, Hyderabad 500007, India. 

