# Supplementary figures and images for: Interaction of Pyrrolobenzodiazepine (PBD) Ligands with Parallel Intermolecular G-Quadruplex Complex Using Spectroscopy and ESI-MS
Source: PLoS One. 2012 Apr 27;7(4):e35920. doi: 10.1371/journal.pone.0035920 (PMC3338766; doi:10.1371/journal.pone.0035920)

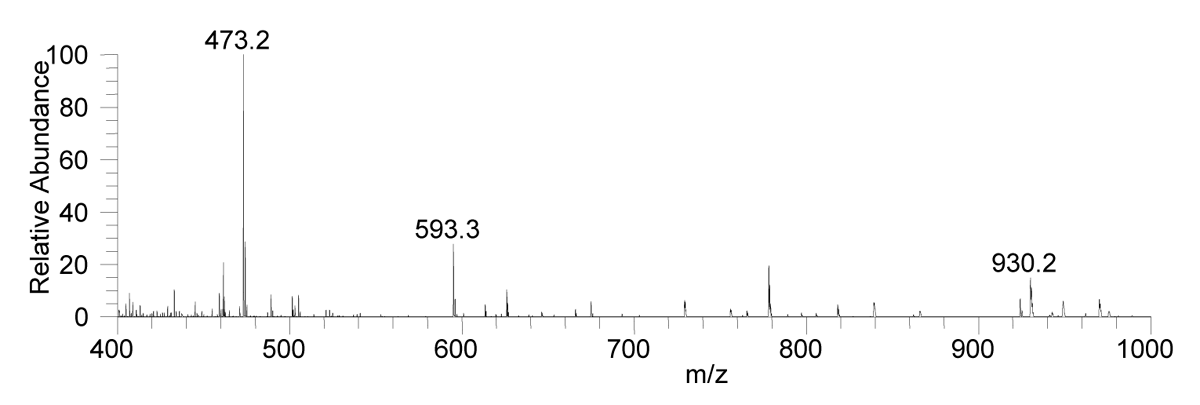

Supplement: Figure S1 — Expanded ESI-MS spectrum (from m/z 400–1000) of d(T2G8) G-quadruplex. (TIF) [file pone.0035920.s001.tif]

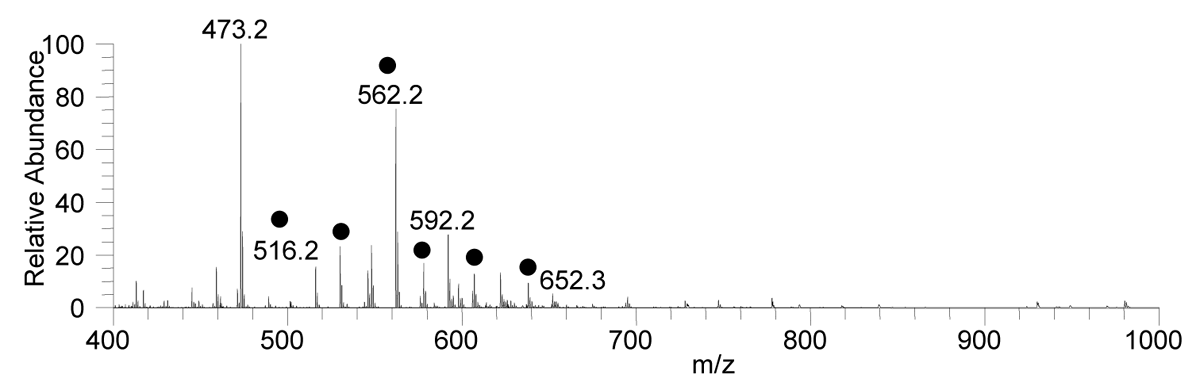

Supplement: Figure S2 — Expanded ESI-MS spectrum (from m/z 400–1000) of d(T2G8) G-quadruplex with PBD1. The interaction peaks were marked with “•”. (TIF) [file pone.0035920.s002.tif]

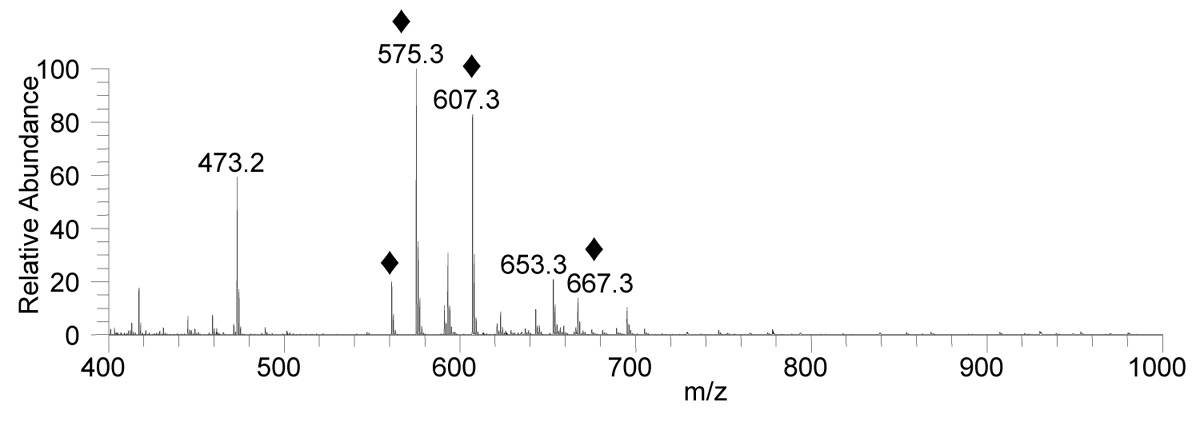

Supplement: Figure S3 — Expanded ESI-MS spectrum (from m/z 400–1000) of d(T2G8) G-quadruplex with PBD2. The interaction peaks were marked with “♦”. (TIF) [file pone.0035920.s003.tif]

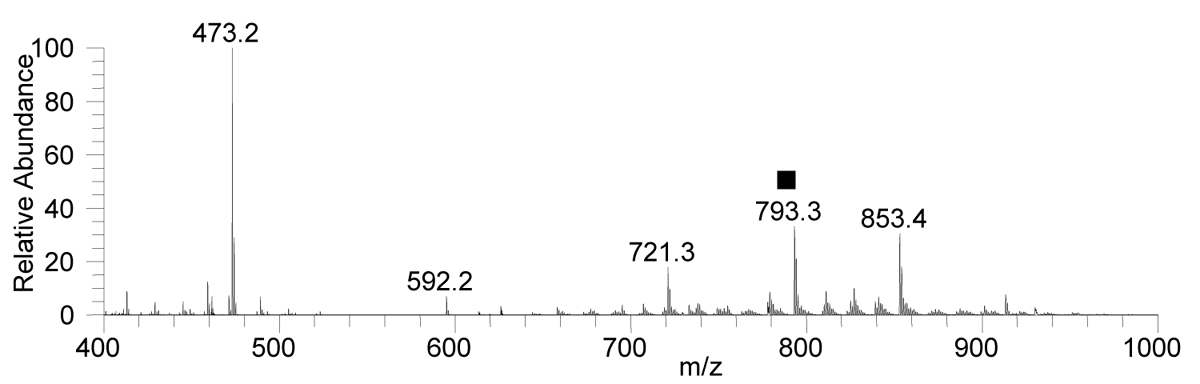

Supplement: Figure S4 — Expanded ESI-MS spectrum (from m/z 400–1000) of d(T2G8) G-quadruplex with TMPyP4. The interaction peaks were marked with “▪”. (TIF) [file pone.0035920.s004.tif]
